# Genomic insights into adaptations to survival and toxicity of cyanobacteria in hot springs: a study comprising cyanobacterial genomes from Europe, Iceland, and Central Asia

**DOI:** 10.7717/peerj.21284

**Published:** 2026-06-03

**Authors:** Nataliia Khomutovska, Agnieszka Rudak, Spyros Gkelis, Mikołaj Kokociński, Iwona Jasser

**Affiliations:** 1 Department of Plant Protection Biology, Swedish University of Agricultural Sciences, Lomma, Sweden; 2 Faculty of Biology, University of Warsaw, Warsaw, Poland; 3 School of Biology, Aristotle University of Thessaloniki, Thessaloniki, Greece; 4 Faculty of Biology, Adam Mickiewicz University of Poznan, Poznań, Poland

**Keywords:** Thermophilic cyanobacteria, Genome sequencing, Extreme environments, Thermal springs

## Abstract

**Aim of the study:**

Genomic resources for thermophilic cyanobacteria remain limited, hindering understanding of their ecology and adaptations to extreme high-temperature environments. This study characterizes the genomic features of thermophilic cyanobacteria from thermal springs, focusing on their strategies to cope with energy limitations and the genetic basis of secondary metabolite production.

**Material and methods:**

We analyzed six newly assembled genomes, and compared them with reference genomes from thermophilic and mesophilic cyanobacteria. Functional gene analysis, genome size comparisons, and biosynthetic gene cluster (BGC) mining were performed to assess adaptations.

**Results:**

Genome sizes varied substantially, with *Hillbrichtia pamiria* showing a notably reduced genome, whereas *Calothrix thermalis* and *Thermoleptolyngbya hindakii* were similar to or larger than related mesophiles. Secondary metabolite BGCs were generally reduced in thermophiles, especially in *H. pamiria*, although larger genomes retained diverse BGCs, including cyanotoxins, nonribosomal peptide synthetases (NRPS), ribosomally synthesized and post-translationally modified peptides (RiPPs) and terpenes. Core functions, the Calvin cycle and nitrogen fixation, were conserved, while lineage-specific variation occurred in Tricarboxylic Acid (TCA) cycle, sulfur assimilation, and denitrification genes. *Amphirytos necridicus* harbors features suggesting adaptation to low-energy environments, like alternative TCA cycle enzymes.

**Conclusions and relevance:**

These findings expand the genomic and functional landscape of thermophilic cyanobacteria, reveal lineage-specific adaptations, and underscore the value of learning from these natural strategies for potential biotechnological applications.

## Introduction

Thermal springs are extreme environments where only highly specialized microorganisms can persist. Among these, thermophilic cyanobacteria stand out as true extremophiles, having evolved unique molecular and genomic adaptations that enable survival and growth at high temperatures conditions that exclude most other life forms. These genomes provide a valuable window into the evolutionary processes that shape life under thermal stress, revealing how extremophiles balance metabolic efficiency, stress resilience, and genomic streamlining. Studying these organisms enhances our understanding of the molecular mechanisms underlying thermotolerance and offers opportunities to harness their unique biochemical and metabolic capacities for biotechnological applications (Ward, Castenholz & Miller, 2012; Kees et al., 2022; Klatt et al., 2011). Adaptation to high temperatures in thermophilic cyanobacteria involves the coordinated action of multiple molecular strategies, including protein stabilization by molecular chaperones, membrane remodeling for thermal resilience, robust DNA repair systems, and enzymatic machinery optimized for heat stress. Together, these adaptations illustrate how extremophilic genomes encode both the flexibility and robustness necessary to thrive under extreme conditions, providing a model for understanding evolution in such environments and for discovering novel biomolecules with industrial and pharmaceutical relevance. Metagenomic analyses of microbial mats have revealed extensive population diversity among thermophilic lineages, as well as unique genomic traits that underpin survival in extreme heat (Pedersen & Miller, 2017; Kees et al., 2022).

### Molecular mechanisms of thermal adaptation

Based on previous studies, thermophilic cyanobacteria maintain cellular function under heat stress through coordinated programmes targeting protein stability, genome integrity, and membrane resilience. Protein denaturation represents a major challenge at elevated temperatures, which is counteracted by the expression of heat shock proteins (Hsp20, Hsp60/GroEL, Hsp70/DnaK) and co-chaperones (DnaJ, GrpE) that prevent aggregation and assist in protein refolding (Wang, Cen & Zhao, 2015; Suzuki, Simon & Slabas, 2006). Membrane integrity is preserved through genes such as *fatB* and desaturases (*des*), which adjust fatty acid composition, and by sulfoquinovosyl diacylglycerol synthesis mediated by *sqdX* and *sqdB* (Sato et al., 2017; Eungrasamee et al., 2024). DNA repair systems—including UvrABC, MutSL, RecA, and Dps proteins—maintain genomic stability under heat stress. Thermophiles also rely on structurally stable metabolic enzymes (*gdhA*, *pyk*, *cox*) and antioxidant systems (SOD, KatG, Trx, Prx) to sustain biochemical reactions at high temperatures (Vieira Vaz et al., 2020; Dietz, 2011). Photosynthetic efficiency is maintained *via* enhanced carbon-concentrating mechanisms (CCM), reflecting the co-evolution of energy capture and thermal adaptation (Brito et al., 2017).

### Genomic markers of thermophily

Based on comparative genomic analyses, several gene families and regulatory systems characterise thermophilic cyanobacteria. These include regulatory genes, including sigma (*σ*) factors, which are proteins that bind RNA polymerase and direct it to specific promoters to control transcription under heat stress (Wang, Cen & Zhao, 2015), thermostable C-phycocyanins (Kees et al., 2022), and gene patterns from metagenome-assembled genomes (MAGs) that differentiate thermophilic lineages (Pedersen & Miller, 2017). Stress-response genes encoding chaperones and transcriptional regulators further define strategies for heat acclimation (Panyakampol et al., 2015).

### Cyanotoxin production in extreme environments

Based on field studies, thermophilic cyanobacteria are capable of producing hepatotoxins (*e.g.*, microcystins) and neurotoxins (*e.g.*, anatoxin, BMAA, DAB, AEG isomers) in hot springs and geothermal lakes (Cirés, Casero & Quesada, 2017; Jablonska et al., 2024). Cyanotoxins in these extreme environments pose ecological risks, including documented wildlife mortality events, such as deaths of Lesser Flamingos in Lake Bogoria, highlighting the environmental and health relevance of thermophilic cyanobacteria.

### Study rationale and objectives

Recent genomic research continues to expand understanding of thermophilic cyanobacterial ecology and adaptation (Jasser et al., 2022). However, genomic resources remain limited for many thermophilic genera, restricting detailed evolutionary and functional analyses. By sequencing and analyzing high-quality genomes from multiple thermophilic habitats across Europe, Iceland, and Central Asia, including previously uncharacterized lineages within the Oscillatoriaceae and FACHB-T130 families, this study provides a foundation for understanding the molecular and evolutionary basis of thermotolerance. These genomes enable detailed comparative analyses of metabolic pathways, regulatory networks and stress-response mechanisms, offering insights into how cyanobacteria adapt to extreme thermal environments and providing resources for future ecological, evolutionary and biotechnological studies. To address this gap, the present study aims to characterize the genomic potential of thermophilic cyanobacteria. Specifically, we pursue:

1. Genome mining to identify genes and pathways essential for thermophilic life.
2. Comparative genomic analysis with related mesophilic and thermophilic strains to identify conserved *versus* unique traits.
3. Exploration of biosynthetic gene clusters (BGCs) to assess potential secondary metabolite production with ecological and biotechnological relevance.

## Materials and Methods

### Strains used for genome sequencing

**Figure 1:**
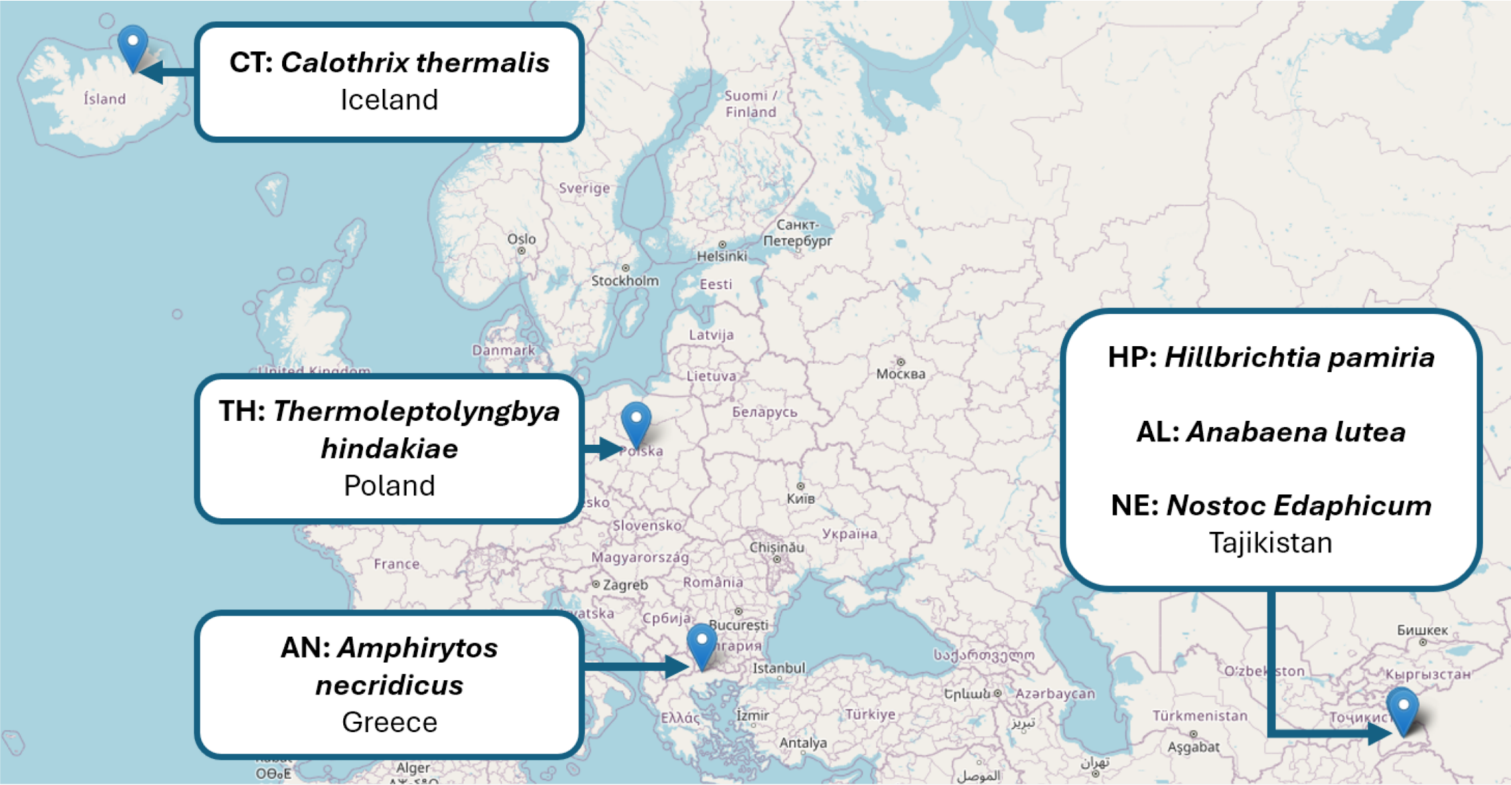
Geographic origin, taxonomic identity and abbreviations of thermophilic cyanobacterial strains which genomes were sequenced and analysed in this study. The analysed strains originate from diverse geothermal environments, including thermal springs in Greece (*Amphirytos necridicus*), geothermal ponds in Poland (*Thermoleptolyngbya hindakiae*), hot springs in Tajikistan (*Hillbrichtia pamiria*, Cylx 15.1), and geothermal baths in Iceland (*Calothrix thermalis*). *A. necridicus*, *T. hindakiae*, *H. pamiria*, and *C. thermalis* were previously isolated, taxonomically described, and characterized with respect to thermotolerance (Jasser et al., 2022). *Anabaena lutea* and *Nostoc edaphicum* were previously reported as members of microbial mats E08 and E11, respectively (Khomutovska et al., 2020). Map data ©OpenStreetMap contributors (CC BY-SA 2.0).

The selected strains originate from locations shown in Fig. 1, with diverse thermal environments, including thermal springs in Greece (*Amphirytos necridicus*), geothermal ponds in Poland (*Thermoleptolyngbya hindakiae*), hot spring habitats in Tajikistan (*Hillbrichtia pamiria, Cylx 15.1*), and from geothermal baths in Iceland (*Calothrix thermalis*). The strains *A. necridicus*, *T. hindakiae*, *H. pamiria,* and *C. thermalis* were previously isolated, taxonomically described, and characterised with respect to thermotolerance in our earlier study (Jasser et al., 2022). *Anabaena lutea* and *Nostoc edaphicum* were not analysed previously but described as members of microbial mats E08 (*A. lutea*) and E11 (*N. edaphicum*) by Khomutovska et al. (2020). Sampling and isolation steps are presented in Fig. S1.

### DNA extraction, genome sequencing, assembly, and annotation

#### Genomic DNA extraction and sequencing

Genomic DNA was extracted from fresh cyanobacterial biomass using the E.Z.N.A.^®^ Soil DNA Kit (Omega Bio-Tek, Norcross, GA, USA), following the manufacturer’s instructions. Sequencing was performed exclusively on DNA from pure isolates; no metagenomic datasets or environmental metagenome-assembled genomes (MAGs) were included in this study. Library preparation and whole-genome sequencing were performed commercially by Eurofins Genomics using the Illumina NovaSeq 6000 platform with a 2 × 150 bp paired-end configuration, targeting a minimum of 100 × average coverage for each genome (up to ∼10 Mb). Raw sequencing data (FASTQ files) are available *via* ENA under project PRJEB93759. In addition, assembled genomes for each species, along with annotation outputs generated in KBase, have been deposited in Zenodo (doi: 10.5281/zenodo.17995636), ensuring full reproducibility of downstream analyses: https://narrative.kbase.us/narrative/222168.

#### Genome assembly and quality control

A standardized genome mining protocol for cyanobacteria was applied (Shaffer et al., 2020; Aziz et al., 2008), ensuring reproducible and comprehensive identification of genomic features. Raw reads were processed using Trimmomatic v0.39 to remove adapters and low-quality sequences, and sequence quality was assessed with FastQC v0.11.9 (Andrews, 2010). Contaminant removal was performed at two levels. First, read-level filtering was conducted using Kraken2 v2.1.2 (Wood, Lu & Langmead, 2019), conservatively applied to remove only reads assigned to well-characterized non-cyanobacterial contaminants (*e.g.*, human or other common laboratory contaminants), thereby minimizing the risk of discarding sequences from poorly represented cyanobacterial lineages. Second, post-assembly filtering was applied, where assembled contigs were screened using Kraken2 and ZEUSS v1.0 (Alvarenga, Fiore & Varani, 2017) to confirm cyanobacterial origin and remove any residual contamination. Genome assembly was performed using SPAdes v3.15.4 (Prjibelski et al., 2020), followed by scaffolding and gap closure with Platanus v1.2.4 (Kajitani et al., 2019). Assembly quality was evaluated using Assemblathon v1.0 (Bradnam et al., 2013) and QUAST v5.0.2 (Mikheenko et al., 2018). These steps, combined with contaminant filtering and assembly polishing, resulted in high-quality genomes suitable for downstream comparative and functional analyses, although a small number of assemblies with lower completeness or higher contamination were retained and interpreted with appropriate caution.

Functional annotation was carried out with DRAM v1.2.4 (Shaffer et al., 2020) and RAST v2.0 (Aziz et al., 2008), enabling systematic identification of genes involved in primary metabolism, stress adaptation, and secondary metabolite biosynthesis. This genome mining approach allowed us to detect both conserved and lineage-specific genomic traits.

#### Comparative genomics and phylogenetic analysis

**Figure 2:**
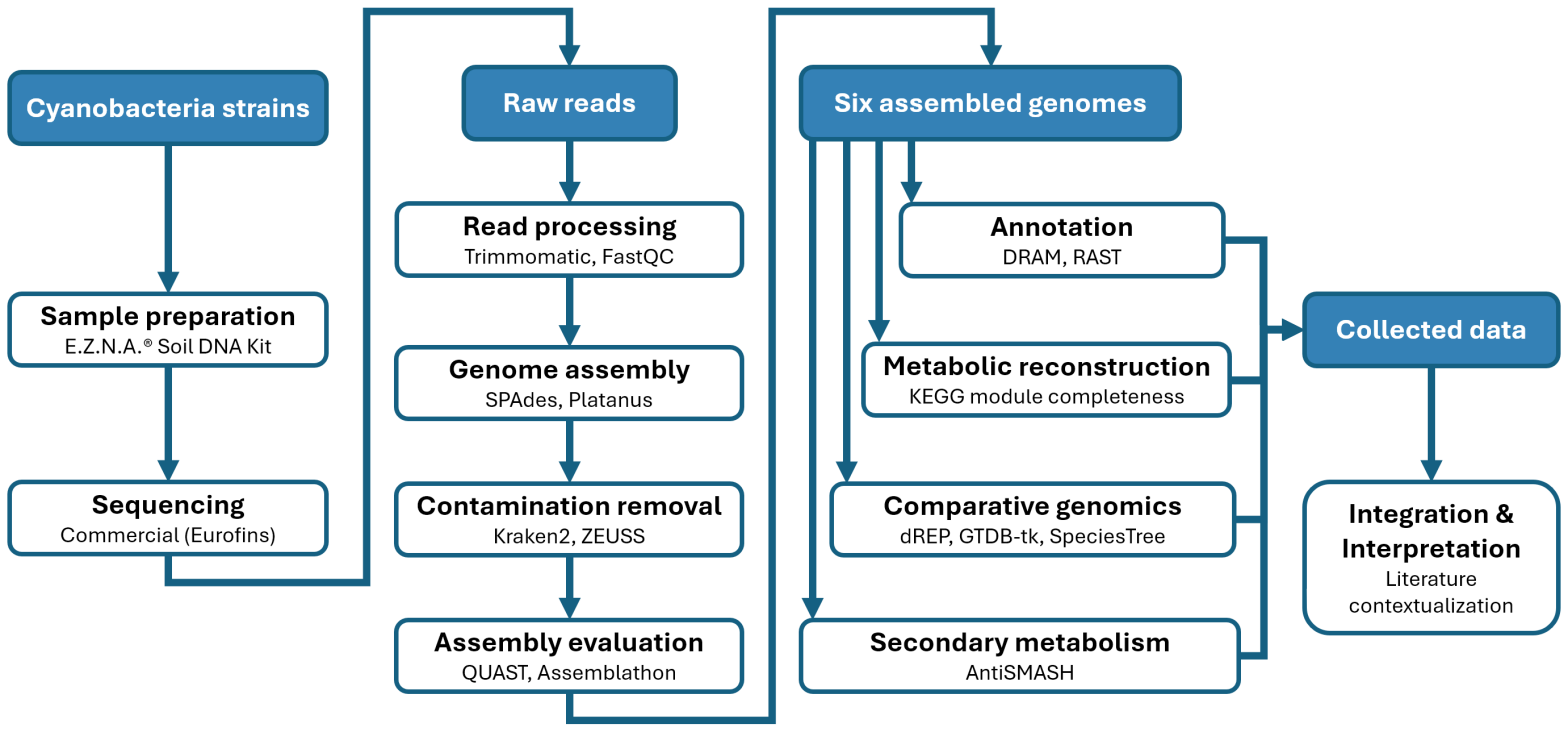
Overview of the genomic analysis workflow and bioinformatic tools used in this study. Workflow summarizing genome assembly, quality assessment, annotation, and comparative genomic analyses. Prior to comparative analysis, a comprehensive survey of the literature and public genomic databases was conducted to identify all available thermophilic cyanobacterial genomes; all relevant genomes were included to enable robust comparison of conserved and unique genomic and metabolic features.

For comparative purposes, additional reference and published genomes were included, selected based on phylogenetic proximity to our isolates, as well as representative thermophilic and mesophilic cyanobacteria. Full list of used genomes is in Table 1. Prior to selection, a comprehensive survey of the literature and public genomic databases was conducted to identify all available thermophilic cyanobacterial genomes, and all relevant genomes available at the time of analysis were included. This allowed robust assessment of conserved *versus* unique genomic features and metabolic capacities. The whole workflow summary is presented in Fig. 2.

**Table 1:** List of additional reference genomes included in our study as comparisons to our assembled genomes.

Reason for inclusion	Organism	GenBank access number	Thermophilic	Source publication
Closest relatives to *Hillbrichtia pamiria* according to phylogenetic tree (Fig. S3)	*Coleofasciculus sp*.	GCF_014698845.1	no	Chen et al. (2021)
	*Cyanosarcina radialis*	GCA_019359615.1	no	Ward et al. (2021)
	*Rippkaea orientalis*/*Synechococcus* sp.	GCF_000021805.1	no	Cornet et al. (2018)
	*Coleofasciculus chthonoplastes*	GCA_000155555.1	no	Tang et al. (2024)
Thermophilic species	*Thermosynechococcus vestitus*	GCA_003555505.2	yes	Liang et al. (2018)
	*Moorella thermoacetica* ATCC 39073	GCF_000013105.1	yes	Redl et al. (2020)
	*Leptodesmis sichuanensis*	GCA_021379005.1	yes	Tang et al. (2024)
	*Leptolyngbya* cf.* ectocarpi*	GCA_015207065.1	no	Tang et al. (2024)
Non-thermofilic species as a reference group	*Leptolyngbya boryana*	GCF_002142475.1	no	Hiraide et al. (2014)
	*Nostoc punctiforme*	GCA_000020025.1	no	Cornet et al. (2018)
	*Nostoc* sp. PCC 7107	GCA_000316625.1	no	Shih et al. (2013)
	*Anabaena* sp. PCC 7108	GCA_000332135.1	no	Shih et al. (2013)
	*Calothrix* sp. PCC 6303	GCF_000317435.1	no	Shih et al. (2013)
	*Calothrix* sp. PCC 7103	GCF_000331305.1	no	Shih et al. (2013)

To evaluate genome similarity and redundancy, dRep v2.6.2 (Olm et al., 2017) was used to enable accurate genomic comparisons and de-replication. Phylogenetic relationships were inferred using GTDB-Tk v2.12.10 (Chaumeil et al., 2022) and validated with the “Insert Set of Genomes Into SpeciesTree” pipeline implemented in FastTree v2.1.10 (Price, Dehal & Arkin, 2010), with reference genomes from GTDB (Parks et al., 2020).

All analyses were conducted within the KBase platform, using integrated modules for read preprocessing, genome assembly (SPAdes), genome annotation (RAST), and comparative genomics workflows. The KBase narrative provides full reproducibility, including parameter settings and intermediate outputs for each step.

## Results

### Assembled genomes

We assembled six genomes of isolated strains with a completeness rate of over 87%. Short contigs with a length of <1,000 bp were removed before the analysis. Assembly statistics are summarized in Table 2, including N50 (73,141–357,711 bp), L50 (7–32), number of contigs (41–385), and largest contig length (297,150–635,415 bp), indicating variable but overall moderate-to-high assembly continuity among the genomes. Detailed assembly and contig statistics are additionally presented in Figs. S2 and S3. Table 2 and Table S1 provide genome codes used throughout the manuscript, together with basic genome characteristics and initial gene presence screening. Genes *nif* D, *nif* K, *nif* H, and Rubisco S and L have been found in all newly assembled genomes. *A. necridicus* and *H. pamiria* genomes had the highest percentage of GC pairs, having 50% and 47%, respectively, with *H. pamiria* also being the smallest genome among our assemblies, having 4,7 mln bp. Other smaller genomes included *A. lutea* with 5,7mln and *T. hindakiae* and *A. necridicus* with 6,6mln bp. Despite its small genome size, *A. lutea* had the lowest GC percentage. Cytochrome oxidase genes have been found in all genomes, while quinol oxidase is also found in *C. thermalis*, *H. pamiria*, *N. edaphicum*, and *T. hindakiae*. All species differed in the presence of sulphur metabolism genes, and nitrate reductase genes *nir*B and *nir*D were found only in two genomes (*A. lutea* and *T. hindakiae*).

**Table 2:** Basic statistics of assembled genomes with completeness and contamination percentage, length of genomes, contigs and presence of Oxygen, Sulfur, and Nitrogen metabolism genes.

**Genome code**	**CheckM** **completeness (%)**	**CheckM contamination (%)**	**Genome size** **(bp)**	**N50 (bp)**	**L50 (bp)**	**Number of contigs**	**LargestContig (bp)**	**G+C** **(%)**	**BioE: Oxygen**	**BioE: Sulfur**	**BioE: Nit_Ox_Red**
AN	87.93	16.80	6,669,676	135,684	17	250	297,150	50.70	coxA, coxB	aprA, sdo	nirB, nirD
CT	96.55	29.94	9,123,886	241,165	12	385	573,875	41.30	coxA, coxB, cydA, cydB	cysC, sat, sqr, sdo	–
HP	99.33	0.67	4,788,368	357,711	7	41	635,415	47.60	coxA, coxB, cydA, cydB	cysC, sat, sqr, sdo	–
TH	90.98	4.80	6,634,043	169,088	10	83	455,092	43.30	coxA, coxB, cydA, cydB	cysC, sat, sqr	nirB, nirD
AL	99.67	0.56	5,763,363	184,907	10	59	426,492	39.30	coxA, coxB	sat	–
NE	99.78	0.44	8,207,313	73,141	32	246	330,772	41.50	coxA, coxB, cydA, cydB,	cysC, sat, sqr, sdo	–

### Phylogenomics

Included additional reference genomes for comparative purposes were selected based on quantitative measures of phylogenetic proximity to our isolates, including average nucleotide identity (ANI) and alignment fraction, as well as representation of thermophilic and mesophilic cyanobacteria. For example, *Amphirytos necridicus* (AN) showed 96.17% ANI and 0.824 alignment fraction with its closest GTDB-Tk placement (*Elainella sp000733415*), while *Calothrix thermalis* (CT) shared 94.35% ANI and 0.904 alignment fraction with *Calothrix sp015272395*. Table 3 contains details of all newly assembled genomes.

**Table 3:** Taxonomic assessment of assembled genomes according to GTDB-Tk. All genomes were assigned as d__Bacteria;p__Cyanobacteriota;c__Cyanobacteriia. According to traditional taxonomy (*e.g.*, classification used within NCBI), these genomes belong to the phylum Cyanobacteria and class Cyanophyceae.

**Genome**	**Taxon**	**Closest placement taxonomy**	**Closest placement ANI**	**Closest placement alignment fraction**	**Taxonomic classification method**	**RED value**
AN	*Amphirytos necridicus*	o__Elainellales;f__Elainellaceae; g__Elainella;s__Elainella sp000733415	96.17	0.824	ANI screen	–
CT	*Calothrix thermalis*	o__Cyanobacteriales;f__Nostocaceae; g__Calothrix;s__Calothrix sp015272395	94.35	0.904	topology and ANI	0.98999
HP	*Hillbrichtia pamiria*	o__Cyanobacteriales;f__FACHB-T130; g__;s__	–	–	topology	0.84051
TH	*Thermoleptolyngbya hindakiae*	o__Cyanobacteriales;f__Oscillatoriacea; g__;s__	–	–	novelty determined using RED	0.85017
AL	*Anabaena lutea*	o__Cyanobacteriales;f__Nostocaceae; g__Anabaena;s__Anabaena lutea	94.37	0.834	topology and ANI	0.9955
NE	*Nostoc edaphicum*	o__Cyanobacteriales;f__Nostocaceae; g__Nostoc;s__Nostoc edaphicum_A	94.94	0.779	topology and ANI	0.99665

AN, HP, and TH are all strains of a newly described genera and/or species (Jasser et al., 2022); therefore, their taxonomic assignment could not be performed fully with existing databases. *Hillbrichtia* appears to be the closest relative to members of the *Coleofasciculus* genus; however, not with a high certainty (Fig. S5). *Amphirytos* genus seems to be closely related to *Elainella* (Table 3). TH is a newly described species of *Thermoleptolyngbya hindakiae,* which belongs to Oscillatoriaceae family and is grouped with *Oscillatoria* genus. CT assignment confirmed our initial assessment of this genome to the species *Calothrix thermalis*. AL and NE genomes have been assigned to the family Nostocaceae (Fig. S6). AL appears to be assigned to *Anabaena* genus and is closely related to *A. lutea*, while NE is assigned to *Nostoc,* exhibiting high similarity to *N. edaphicum* (Table 3). Full phylogenetic trees are shown in Figs. S4 and S6. The genomes of *Amphirytos necridicus* (AN) and *Calothrix thermalis* (CT) are draft-quality, with 87.93% completeness and 16.8% contamination (AN) and 29.94% contamination (CT). These genomes were included in analyses for completeness of representation but interpreted cautiously.

Figure S4 shows a phylogenomic tree compatible with NCBI taxonomy (based on http://algaebase.org (Guiry & Guiry, 2025)), illustrating the phylogenetic relationships among the cyanobacterial genomes included in this study. Genomes highlighted in blue correspond to the isolates sequenced in this study, as well as thermophilic reference genomes included for comparative purposes. This visualization confirms the placement of our sequenced strains within their respective phylogenetic clades and allows comparison with representative thermophilic cyanobacteria.

### Functional genes analysis

CheckM analysis shows that most genomes are highly complete (>96%) with low contamination (<1%). The exceptions are *Amphirytos necridicus* (∼88% completeness, 16.8% contamination) and *Calothrix thermalis* (∼96.6% completeness, 29.9% contamination). Reduced completeness and higher contamination in these genomes may partly explain some of the observed differences in metabolic modules. All genomes are presented with full quality metrics to allow readers to interpret functional annotations appropriately in comparative analyses.

**Figure 3:**
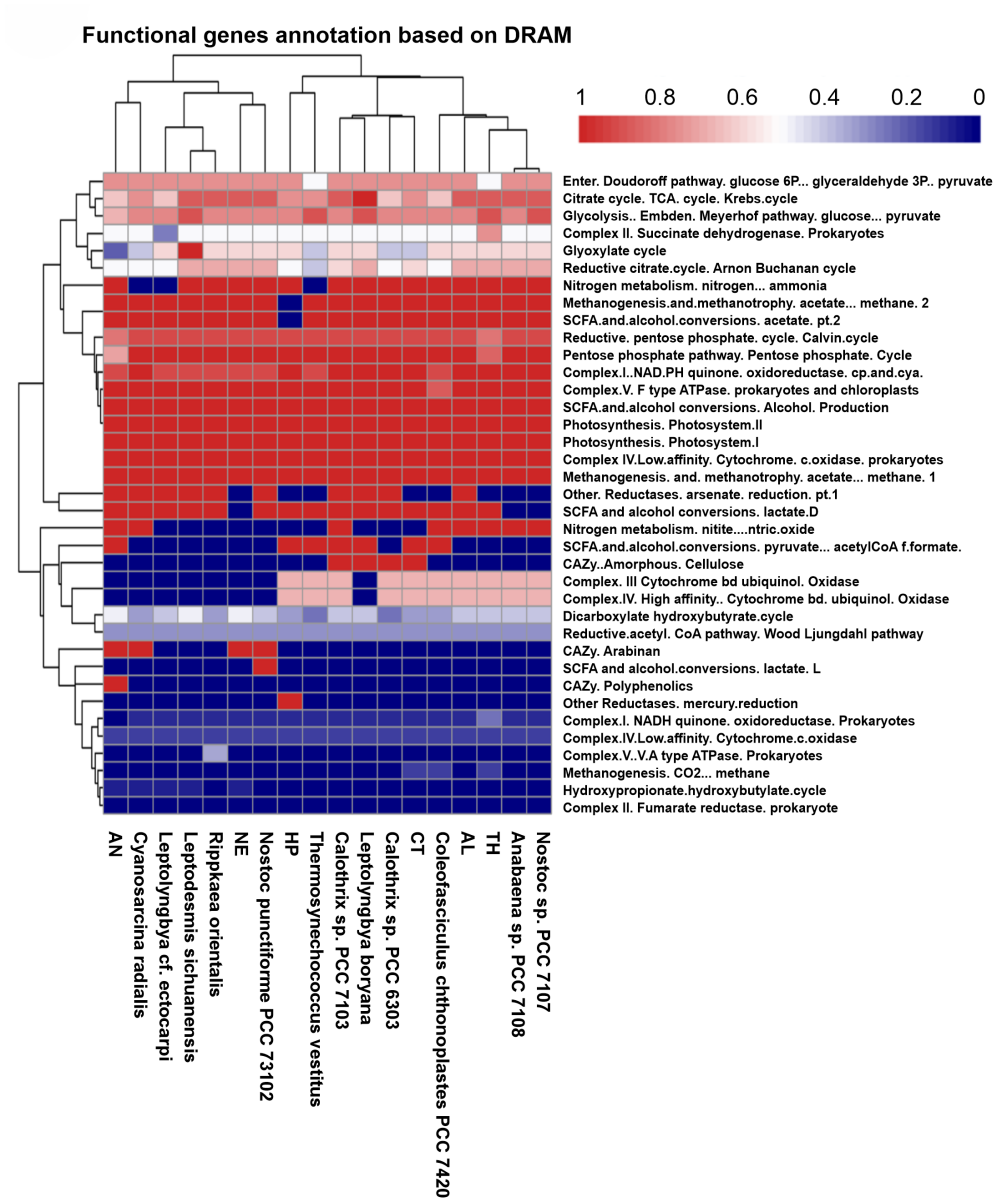
Heatmap showing DRAM-based metabolic annotation and pathway completeness across analysed cyanobacterial genomes. Completeness scores of major metabolic pathways based on DRAM annotation. All genomes show high completeness of the Calvin–Benson cycle, confirming conserved CO_2_ fixation capacity. Variability is observed across other pathways, including the tricarboxylic acid (TCA) cycle, glycolysis, the pentose phosphate pathway, and alternative carbon-fixation pathways, indicating differences in metabolic strategies and potential adaptations to thermophilic environments.

Figure 3 presents a heatmap summarizing DRAM-based functional annotations across the studied cyanobacterial genomes. Each row represents a genome, and columns correspond to metabolic modules or pathways, including carbon fixation, central carbon metabolism, respiratory complexes, nitrogen and sulfur metabolism, photosynthesis, and carbohydrate-active enzymes (CAZymes). Values indicate the completeness or presence of each pathway/module, allowing a visual comparison of conserved *versus* variable functions among the strains. Only thermophilic strains are included in the heatmap for direct functional comparison.

All studied genomes conserve the Calvin-Benson cycle (reductive pentose phosphate pathway) with near-complete completeness (∼1.0), reaffirming its central role in CO_2_ fixation across cyanobacteria (Fig. 3). However, key differences emerge in other carbon metabolic routes. The tricarboxylic acid (TCA) cycle, vital for energy generation and biosynthesis, is most complete in *L. boryana*, indicating strong aerobic respiration capacity. In contrast, *A. necridicus* and *Calothrix* sp. PCC 6303 shows reduced TCA completeness (∼0.63–0.75), implying alternative metabolic strategies potentially linked to thermophilic adaptation. Glycolysis *via* the Embden-Meyerhof-Parnas pathway is well developed in *Leptodesmis sichuanensis*, accompanied by an active pentose phosphate pathway, which supports biosynthesis through NADPH production.

Alternative carbon fixation pathways show varied representation: the reductive citrate (reverse TCA) cycle is prominent in *Anabaena* sp. PCC 7108 and *Calothrix* sp. PCC 7103 (∼0.6–0.7) (Fig. 3), enabling utilization of diverse carbon sources, while *A. necridicus* lacks significant activity here but shows traces of the hydroxypropionate-hydroxybutylate cycle, suggesting limited alternative CO_2_ fixation. The Wood-Ljungdahl pathway, an energy-efficient autotrophic route, is notably enriched in *L. boryana* and *L. sichuanensis*.

Photosystems I and II are universally conserved and nearly complete across most genomes, especially in *L. boryana*, *Anabaena* sp. PCC 7108, and *Calothrix* sp. PCC 7103 (Fig. 3), reinforcing their phototrophic lifestyle. Conversely, *A. necridicus* exhibits a reduced presence of photosystem components, indicative of a possible mixotrophic or heterotrophic strategy. The respiratory electron transport chain is well-developed in *L. sichuanensis* and *L. boryana*, with robust complexes I and III, facilitating efficient ATP production under variable environmental conditions.

Nitrogen fixation genes are conserved in *Anabaena* sp. PCC 7108 and *Calothrix* sp. PCC 7103 (Fig. 3), supporting their key ecological role in nitrogen cycling. In contrast, *A. necridicus* shows minimal nitrogen fixation potential, possibly relying on external nitrogen sources. Sulfur metabolism genes for dissimilatory sulfate reduction are uniquely present in *A. necridicus*, suggesting adaptation to sulfur-rich environments and distinguishing it metabolically from other species.

Carbohydrate-active enzymes (CAZymes), essential for polysaccharide degradation, are abundant in *L. sichuanensis* and *L. boryana*, enabling efficient breakdown of cellulose, starch, and pectin, which may contribute to ecological versatility and biotechnological applications. *A. necridicus*, however, lacks key enzymes such as chitinase and xylanase, limiting its polysaccharide degradation capacity. Furthermore, short-chain fatty acid metabolism, including acetate and butyrate fermentation, is well-developed in *L. sichuanensis* and *Calothrix* sp. PCC 7103, supporting survival in fluctuating oxygen conditions (Fig. 3).

To better understand the metabolic potential of the cyanobacterial strains, genome-scale metabolic models (GEMs) were constructed in KBase for six representative genomes: *A. necridicus*, *C. thermalis*, *N. edaphicum*, *A. lutea*, *T. hindakiae*, and *H. pamiria*. The models vary in size, with *C. thermalis*, belonging to Nostocales, showing the most extensive metabolic network (1,141 reactions and 1,195 compounds), indicating broader biosynthetic and catabolic versatility. NE and AL, also belonging to Nostocales, have moderately large models (559 and 570 reactions, respectively), consistent with the typically more complex life strategies of this group, including nitrogen fixation and developmental plasticity.

In contrast, *A. necridicus* and *T. hindakiae*, affiliated with Synechococcales and Oscillatoriales, respectively, have smaller models (∼548–549 reactions), reflecting potentially more specialized or streamlined metabolic systems adapted to thermophilic environments. All models share a common structure with two cellular compartments and one biomass objective function. Only minor gap-filling (two reactions per model) was needed to restore model completeness. Full raw data and sequences are deposited under ENA project number PRJEB93759 (Table S2) and DOI: 10.5281/zenodo.17995635.

### Biosynthetic and stress-responsive genes

To investigate the biosynthetic potential and stress adaptation mechanisms of thermophilic cyanobacteria, we performed comprehensive gene screening using the antiSMASH bacterial version to identify secondary metabolite biosynthetic gene clusters, and the MultiGene BLAST application within KBase to screen for functional genes associated with DNA repair, stress tolerance, and metabolic adaptation. The BLAST queries specifically targeted genes involved in DNA repair pathways (*e.g.*, *uvrA*, *uvrB*, *recA*, *mutS*), oxidative stress responses (*e.g.*, *sod*, *katG*, *trx*, *prx*), and thermal stress adaptations such as chaperone systems (*dnaK*, *groEL*, *hsp20*) and membrane stability (*e.g.*, *fatB*, *des*, *sqdX*, *sqdB*). However, the identification of these functional genes was limited by the composition of the reference database. Inspection of best matches showed that most homologous sequences corresponded to non-cyanobacterial thermotolerant organisms, whereas thermophilic or thermotolerant cyanobacteria represented only a small fraction (a few percent) of hits. This highlights the strong taxonomic bias in current genomic resources and emphasizes the need for additional cyanobacterial genome assemblies to better evaluate thermotolerance and stress-response capabilities.

A detailed list of secondary metabolite genes found is in Table S3. In the genomes of NE, AL, CT, and TH, we identified more gene clusters involved in secondary metabolite production than in AN and HP. This aligns with genome size, except for AL, which has many secondary metabolite genes and a considerably smaller genome than those of other closely related taxa. We found genes encoding for terpenes and nonribosomal peptide synthetases (NRPS)-like metabolites in all genomes. Heterocyte glycolipids have been found in both Nostocaceae genomes and in AN.

The BLASTn (KBase) analysis identified the Excinuclease ABC subunit C, a key DNA repair enzyme, in *Calothrix thermalis* (CT) with 75.8% identity over 99.6% alignment coverage (1,897 bp), a highly significant *E*-value of 0.0, and a bit score of 931. In *Nostoc edaphicum* (NE), the same gene was detected with 80.7% identity, but lower alignment coverage (51.0%), with an *E*-value of 0.0 and a bit score of 750. No significant hits were obtained in other genomes, suggesting limited conservation or high sequence divergence across the thermophilic strains. Similarly, ATP synthase alpha chain (EC 3.6.3.14) was found only in NE, showing 81.8% identity over 98.6% alignment coverage (1,499 bp). The presence of mismatches and gaps further indicates potential genetic divergence, limiting direct comparisons among the thermophilic isolates. Overall, BLAST-based screening presented in Table 4, for stress-response and cyanotoxin-related genes is constrained by the scarcity of thermophilic cyanobacterial reference genomes. While Excinuclease ABC subunit C was detected in two genomes, its absence in most other strains prevents strong conclusions regarding conservation. Therefore, we interpret these findings cautiously, highlighting only that the gene is present in some thermophilic isolates rather than asserting a broadly conserved role.

**Table 4:** List of toxin-related and thermophilic-associated genes identified in assembled genomes using BLAST.

**Taxon**	**Gene**	**Related product**	**N** **E**	**AL**	**H** **P**	**CT**	**TH**	**AN**	**Source publication**
*Anabaena* sp.	*mcyA-J*	microcystin	*						Dreher et al. (2019)
*Phormidium*	*mcy* cluster	microcystin	*						Shishido et al. (2019)
*Microcystis aeruginosa*	*uma1-6* cluster	microcystin	*	*					Tillett et al. (2000)
*Leptolyngbya* sp.	*mcyE*	microcystin							Saran & Mishra (2015)
*Prochlorococcus marinus*	*psbA*	photosystem component		*		*	*		Lindell et al. (2004)
*Moorea producens*	NRPS/PKS cluster			*		*			Boudreau et al. (2015)
*Nostoc* sp.	*res* B-like protein, ABC transporter, nostophycin		*	*					Fewer et al. (2011)
*Nostoc punctiforme*	*aerA*	aerolysin							Tikhonova et al. (2017)
*Oscillatoria* sp.	*cyr* cluster	cylindrospermopsin	*			*	*		Mazmouz et al. (2010)
*Desmonostoc muscorum*	*nosA-G*	nostopeptolide	*	*					Mares et al. (2020)
*Desmonostoc muscorum*	*mcxA-C*	muscotoxin	*	*					Mares et al. (2020)
*Nostoc flagelliforme* *Kamptonema laetevirens*	C-phycocyanin beta and alpha subunit			*		*		*	Jiao et al. (2010); Shakena Fathima et al. (2023)
*Fisherella thermalis*	*uvrA*	excinuclease A subunit	*			*			Dagan et al. (2013)

**Notes.**

The “Gene” column reports standard gene names, while the “Related product” column lists the corresponding protein or functional product. In case of whole clusters, the columns are merged by the name of final product. Marks (*) indicate the presence of a gene or cluster in each genome. Source publications are provided for reference.

## Discussion

Comparative genomic analysis of six thermophilic cyanobacterial genomes revealed key adaptive features and metabolic diversity. Most genomes showed completeness levels suitable for broad comparative analyses. For genomes initially below the completeness threshold, quality was improved using standardized bioinformatic procedures, including contaminant removal, scaffolding and gap closure, and assembly polishing, as described in the Methods. To contextualize these genomes, we compared them with closely related reference strains identified through ANI and phylogenomic placement. Several grouped with known taxa such as *Elainella* spp., *Calothrix* spp., *Anabaena* spp., and *Nostoc* spp., while others (TH and HP) appear to represent previously uncharacterized lineages within Oscillatoriaceae and an unclassified FACHB-T130-associated family.

### Genome size, GC content, and general features

Genome sizes ranged from 4.7 Mbp (HP) to 9.1 Mbp (CT), reflecting variation in genome complexity. Larger genomes, such as those of CT and NE (>8.5 Mbp), are consistent with previously described Nostocaceae genomes, which are known for their expansive gene content and secondary metabolite pathways (Huo et al., 2021; Moten et al., 2020). GC content varied from ∼39% (AL) to ∼50% (HP and AN), potentially contributing to thermal genome stability and aligning with ranges reported in thermophilic cyanobacteria (Tang et al., 2022; Vieira Vaz et al., 2020). Two of the assembled genomes, *Amphirytos necridicus* (AN) and *Calothrix thermalis* (CT), are draft-quality, with 87.93% completeness and 16.8% contamination (AN) and 29.94% contamination (CT). These genomes were included in comparative analyses to provide a broader representation of thermophilic cyanobacterial diversity. However, due to incompleteness and contamination, any pathway absences or variations observed in these genomes may partly reflect assembly gaps rather than true biological differences. Consequently, conclusions regarding metabolic adaptations and lineage-specific features are drawn primarily from the more complete, lower-contamination genomes, while observations from AN and CT should be interpreted with caution. This transparent reporting ensures acknowledging both the potential insights and limitations of these draft genomes.

### Respiration and central carbon metabolism

Genes encoding components of the electron transport chain, including cytochrome oxidases (coxA, coxB) and cytochrome bd oxidase (cydA, cydB), were widely distributed. However, AL and AN strains lack cyd genes, indicating potential functional divergence in aerobic respiration (Winstedt et al., 1998). Sulfur assimilation genes (cysC, sat, sqr, sdo) showed heterogeneous presence, reflecting adaptation to local sulfur availability (Yarbrough, 2020). The Calvin cycle (RuBisCO-mediated carbon fixation) was nearly complete (∼1.0) across all genomes, consistent with core carbon fixation pathways conserved in cyanobacteria (Shi & Falkowski, 2008; Shih et al., 2013). The tricarboxylic acid (TCA) cycle exhibited lineage-specific variation: it was incomplete in *A. necridicus* (∼0.63) but largely complete in *Leptolyngbya* and Nostoc-related genomes (0.88–1.0). This partial TCA cycle in some thermophiles reflects a known prokaryotic adaptation: cyanobacteria lack 2-oxoglutarate dehydrogenase, but the cycle can be functionally completed *via* alternative enzymes (Shih et al., 2013; Alcorta et al., 2020). Such streamlining likely supports energy-efficient respiration under thermal stress while maintaining essential metabolite fluxes. Alternative carbon fixation pathways, including the Wood-Ljungdahl and hydroxybutyrate cycles, were absent or incomplete, in agreement with prior observations that these pathways are rare in cyanobacteria (Ragsdale & Pierce, 2008). DRAM-based pathway completeness revealed that some draft genomes, including AN and CT, show reduced completeness for the TCA cycle (∼0.63–0.75). Because these genomes are incomplete or have moderate contamination, these observations may partly reflect assembly gaps rather than true biological differences. Consequently, interpretations regarding alternative metabolic strategies are presented with caution, focusing on high-quality genomes for robust conclusions.

### Nitrogen metabolism

All genomes retained complete nitrogen fixation gene sets (nifHDK), supporting N_2_ → NH_3_ conversion even under thermophilic conditions. The presence of additional nif genes (nifENB) was verified where detectable, ensuring coverage of the minimal set proposed for functional nitrogenase activity (Dos Santos et al., 2012). The nitrite-to-nitric oxide module (nirB/nirD) exhibited variable presence: it was absent in *Hillbrichtia* and *Thermoleptolyngbya*, suggesting differential denitrification capacities among thermophiles, consistent with freshwater cyanobacteria studies (Meeks & Elhai, 2002; Sivonen & Jones, 1999). Nostocaceae genomes (NE and AL) contained additional genes for heterocyte glycolipid biosynthesis, indicating the presence of functional heterocyte-like structures that facilitate oxygen-tolerant nitrogen fixation (Bauersachs et al., 2013).

### Photosynthesis and carbon-concentrating mechanisms

Photosystems I and II were universally present and nearly complete, even in genomes with streamlined metabolic pathways, emphasizing their indispensable role in light-driven energy capture. Some genomes, such as those of *Leptolyngbya spp.,* exhibited an expanded repertoire of photosystem II components and carbon-concentrating mechanisms (CCMs), consistent with adaptations to high-light and carbon-rich environments (Brito et al., 2017). Conversely, *A. necridicus* exhibited reduced photosystem complexity alongside genes supporting heterotrophic carbon utilization, reflecting adaptation to low-energy niches (Pierpont et al., 2024; Tang et al., 2024).

### Secondary metabolism and biosynthetic gene clusters

BGC abundance is generally correlated with genome size. NE, AL, CT, and TH contained more BGCs, whereas AN and HP exhibited reduced secondary metabolite potential. Notably, AL retained numerous BGCs despite a smaller genome, illustrating genome streamlining with selective retention of biosynthetic genes, a phenomenon documented in cyanobacteria from extreme environments (Weber et al., 2015; Shih et al., 2013; Konstantinou et al., 2021). All genomes contained terpene and NRPS-like pathways, reflecting the widespread biosynthetic potential of cyanobacteria (Kehr, Gatte Picchi & Dittmann, 2011).

### Toxin biosynthesis

Microcystin (mcy) clusters were restricted to Nostocaceae genomes, consistent with prior studies (Sivonen & Jones, 1999; Rantala et al., 2006). Cylindrospermopsin (cyr) clusters were detected in *Nostoc edaphicum*, *Calothrix thermalis*, and *Thermoleptolyngbya hindakii*, in line with reports of thermophilic and freshwater cyanobacteria producing this toxin (Mihali et al., 2008). *A. necridicus* and *H. pamiria* lacked toxin genes, correlating with lower secondary metabolite content. Additional ribosomally synthesized and post-translationally modified peptides (RiPPs) and lanthipeptides in TH and AL suggest antimicrobial potential, consistent with the known pharmaceutical relevance of cyanobacterial RiPPs (Arnison et al., 2013). Cyanobactins and microviridins identified in NE and TH highlight further bioactive compound potential (Sivonen et al., 2010) (Table 4).

Some apparent absences in metabolic pathways in AN and CT may reflect incomplete assembly or contamination rather than true biological loss. Therefore, interpretations of metabolic streamlining or alternative strategies are drawn primarily from more complete, low-contamination genomes, whereas draft genomes are included to represent thermophilic diversity.

Importantly, recent population genomics of the thermophilic *Thermosynechococcus taiwanensis* revealed within-species divergence into distinct populations and selective sweeps targeting different sets of genes related to photosynthesis, motility, and ion transport, providing direct evidence of fine-scale adaptation processes in hot spring cyanobacteria. These findings illustrate that even within a single thermophilic species, evolutionary pressures can drive genomic divergence and candidate adaptation at subspecies levels, emphasizing the role of selection in structuring genetic diversity in extreme environments (Chang et al., 2024).

Collectively, these observations demonstrate that thermophilic cyanobacteria balance genomic streamlining with selective retention of adaptive pathways, shaping metabolic, respiratory, and stress-response traits that support survival under thermal, energetic, and ecological constraints. Understanding the mechanisms and evolutionary trajectories that enable cyanobacteria to thrive at high temperatures is crucial for reconstructing how life expands ecological tolerance limits and for uncovering the rules of genome evolution under extreme stress. However, the limited availability of high-quality thermophilic cyanobacterial genomes continues to constrain comprehensive evolutionary and functional analyses. Expanding these genomic resources and dissecting the molecular basis of thermophily will not only advance our understanding of extremophile evolution and ecology but also reveal natural adaptations with tangible biotechnological applications.

These observations are consistent with broader comparative genomic studies showing that late-branching cyanobacterial lineages typically exhibit expanded biosynthetic gene cluster (BGC) repertoires, particularly NRPS and polyketide synthase (PKS) clusters (Shih et al., 2013). For example, Shih et al. (2013) identified over 450 NRPS and PKS gene clusters across 89 cyanobacterial genomes, and Konstantinou et al. (2021) reported numerous BGCs across more than 70 marine Oscillatoriales genomes, underscoring the phylum’s biosynthetic potential. NRPS-like and Type I PKS clusters in CT and NE support the likelihood of bioactive compound synthesis in these strains (Kehr, Gatte Picchi & Dittmann, 2011).

Furthermore, lanthipeptide and ribosomally synthesized and post-translationally modified peptide (RiPP)-like clusters in TH and AL genomes suggest antimicrobial capabilities, which aligns with previous findings on the pharmaceutical relevance of cyanobacterial RiPPs (Arnison et al., 2013). The detection of cyanobactins and microviridins in NE and TH, known for their bioactive properties, further highlights the pharmaceutical potential of these thermophilic strains (Sivonen et al., 2010).

Our results emphasize that, due to the very limited number of publicly available thermophilic cyanobacterial genomes, genome mining and detailed analysis of specific adaptations to extreme environments remain constrained (Shih et al., 2013; Pierpont et al., 2024). Despite these limitations, the present study provides valuable insights into the metabolic and biosynthetic potential of thermophilic cyanobacteria, offering important genomic resources that will facilitate future comparative and functional studies in this underexplored group (Meeks & Elhai, 2002; Weber et al., 2015).

## Conclusions

This comparative genomic study of six thermophilic and mesophilic cyanobacteria reveals substantial diversity in genome size, gene content, and metabolic capabilities that reflect adaptive evolution to extreme thermal environments. Despite this variation, all genomes preserve core functions —including the Calvin cycle and nitrogen fixation—underscoring their essential roles in primary productivity and nitrogen cycling.

Lineage-specific differences in TCA cycle completeness, sulfur assimilation, and denitrification genes illustrate metabolic streamlining consistent with thermophilic strategies for energy conservation and stress resilience. Incomplete TCA cycles in some strains may be compensated by alternative enzymatic routes that enable maintenance of redox balance and energy production under thermal stress. Larger genomes, such as those of *Nostoc*- and *Calothrix*-related strains, encode expanded biosynthetic gene clusters (BGCs) for toxins, NRPS, RiPPs and terpenes, highlighting an enriched secondary metabolism that may contribute to stress mitigation in extreme habitats and offer biotechnological potential. The identification of novel lineages within Oscillatoriaceae and FACHB-T130 families further expands known thermophilic cyanobacterial diversity (Table S4). These newly sequenced genomes complement prior characterizations of hot-spring genera and species, providing a foundation for understanding the genetic basis of thermotolerance, which involves heat shock proteins, regulatory networks, and secondary metabolite pathways shaped by both vertical inheritance and horizontal gene transfer. Recent population genomic studies demonstrate that even within a single species, selective sweeps and localized genomic divergence contribute to adaptation to thermal niches, highlighting ongoing evolutionary processes. By increasing the number of high-quality thermophilic cyanobacterial genomes, this work not only enriches reference datasets for comparative analyses but also advances our understanding of extremophile evolution, ecological adaptation, and the molecular mechanisms underlying thermophily. Furthermore, these insights may guide the discovery of natural adaptations with potential biotechnological applications, reinforcing the broader relevance of expanding genomic resources in this field.

##  Supplemental Information

10.7717/peerj.21284/supp-1 Supplemental Information 1 Appendix S1 containing supplementary tables and figures

10.7717/peerj.21284/supp-2 Supplemental Information 2 Full sequences of 6 newly assembled genomes in the study

## Additional Information and Declarations

### DNA Deposition

The following information was supplied regarding the deposition of DNA sequences: The sequence data is available in the Supplementary File and at ENA: PRJEB93759.

### Data Availability

The following information was supplied regarding data availability: The raw data and sequences are available at Zenodo: - Khomutovska, N., Ruda, A., Gkelis, S., Kokociński, M., & Jasser, I. (2025). Genomic insights into hot spring cyanobacteria [Data set]. Zenodo. https://doi.org/10.5281/zenodo.17995636. The sequence data is available in the Supplemental File and at ENA: PRJEB93759.
