# Supplementary material for: Genomic insights into adaptations to survival and toxicity of cyanobacteria in hot springs: a study comprising cyanobacterial genomes from Europe, Iceland, and Central Asia
Source: PeerJ. 2026 Jun 3;14:e21284. doi: 10.7717/peerj.21284 (PMC13242192; doi:10.7717/peerj.21284)
Supplement: Supplemental Information 1 [file peerj-14-21284-s001.pdf]

Appendix S1

Title: Genomic insights into adaptations to survival and toxicity of cyanobacteria in hot springs: A study comprising cyanobacterial genomes from Europe, Iceland, and Central Asia

Authors:

Nataliia Khomutovska[1,2] (https://orcid.org/0000-0001-6047-2753) n.khomutovska@uw.edu.pl

Agnieszka Rudak[1] (https://orcid.org/0000-0002-9638-7726) a.rudak@uw.edu.pl corresponding author

Spyros Gkelis[3] (https://orcid.org/0000-0002-7746-3199) sgkelis@bio.auth.gr

Mikołaj Kokociński[4] (https://orcid.org/0000-0002-0552-9769) mikolaj.kokocinski@amu.edu.pl

Iwona Jasser[1] (https://orcid.org/0000-0003-0401-1463) i.jasser@uw.edu.pl

[1] *Faculty of Biology, University of Warsaw, Warsaw, Poland*

[2] *Department of Plant Protection Biology, Swedish University of Agricultural Sciences, Lomma, Sweden*

[3] *School of Biology, Aristotle University of Thessaloniki, Thessaloniki, Greece*

[4] *Faculty of Biology, Adam Mickiewicz University in Poznań, Poznań, Poland*

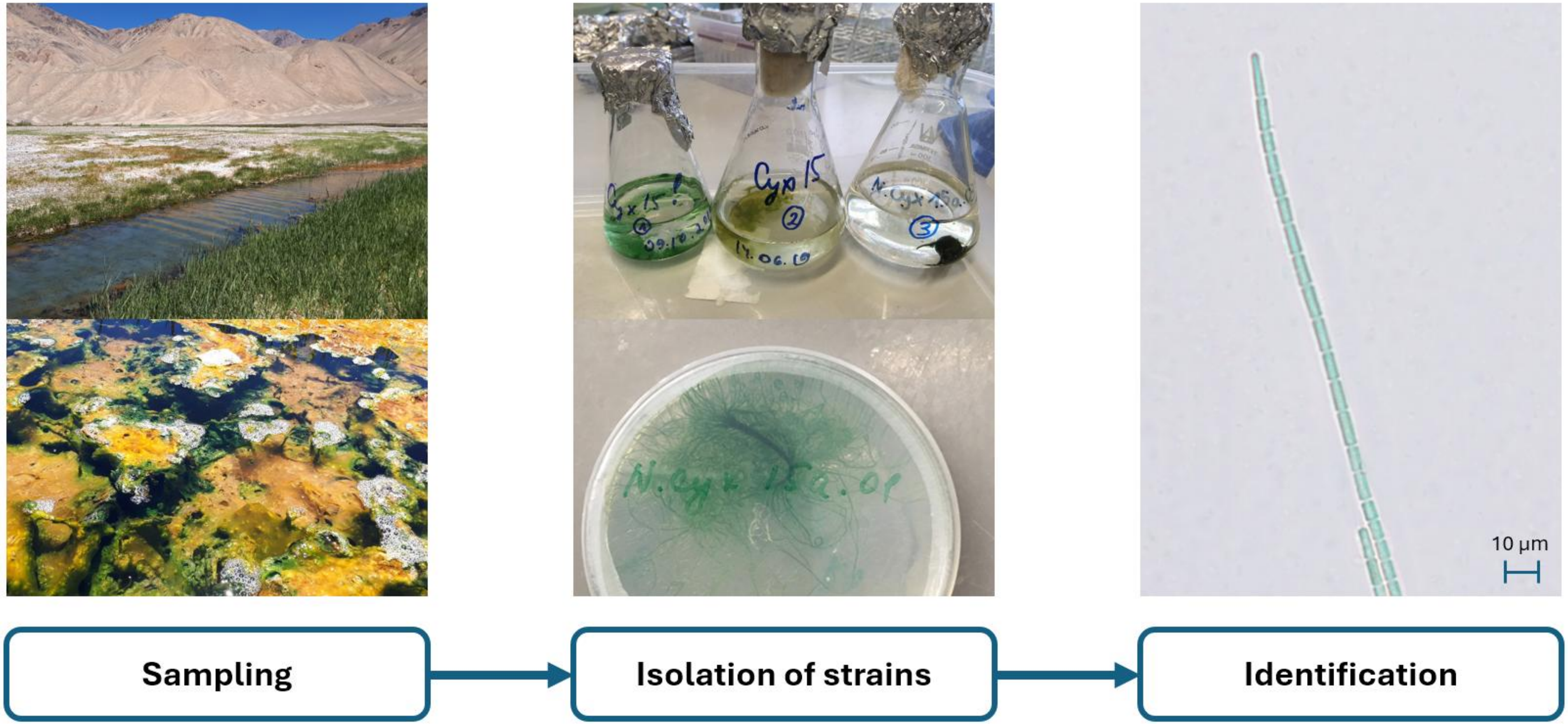

Figure S1: Workflow chart describing preparation and isolation of strains sampled in the course of this study. Each step is illustrated by relevant photography (photos by N. Khomutovska)

Table S1. Comparison of basic genome statistics and initial gene assessment with BioE of all genomes included in the study

Genome	CheckM Completeness (%)	CheckM Contamination (%)	Genome Size (bp)	G+C (%)	N fixation	C_fixation	Oxygen	Sulfur	Nit_Ox_Red
AN – *Amphirytos necridicus*	87.93	16.8	6669676	50.7	nifD,nifK ,nifH	Rubisco-S, Rubisco-L	coxA,coxB	aprA,sdo	nirB,nirD
CT – *Calothrix thermalis*	96.55	29.94	9123886	41.3	nifD,nifK ,nifH	Rubisco-S, Rubisco-L	cydA,cydB,coxA,coxB	cysC,sat,sqr,sdo	-
HP – *Hillbrichtia pamiria*	99.33	0.67	4788368	47.6	nifD,nifK ,nifH	Rubisco-S, Rubisco-L	cydA,cydB,coxA,coxB	cysC,sat,sqr,sdo	-
AL – *Anabaena lutea*	99.67	0.56	5763363	39.3	nifD,nifK ,nifH	Rubisco-S, Rubisco-L	coxA,coxB	sat	-
NE – *Nostoc edaphicum*	99.78	0.44	8207313	41.5	nifD,nifK ,nifH	Rubisco-S, Rubisco-L	cydA,cydB,coxA,coxB	cysC,sat,sqr,sdo	-
TH – *Thermoleptolyngbya hindakiae*	90.98	4.8	6634043	43.3	nifD,nifK ,nifH	Rubisco-S, Rubisco-L	cydA,cydB,coxA,coxB	cysC,sat,sqr	nirB,nirD
*Anabaena_sp. PCC_7108 GCF_000332135.1_*	99.63	0.07	5886741	38.77	nifK,nifH	Rubisco-S, Rubisco-L	cydA,cydB,coxA,coxB	cysC,sat,sdo	-
*Coleofasciculus_chthonoplastes PCC_7420 GCF_000155555.1_*	98.93	0.96	8679041	45.29	nifD,nifK ,nifH	Rubisco-S, Rubisco-L	cydA,cydB,coxA,coxB	cysC,sat,sqr,sdo	-
Coleofasciculus_sp GCA_014698845.1	100	0.37	6198536	46.7	-	Rubisco-S, Rubisco-L	coxA,coxB	cysC,sat	nirB,nirD
Leptodesmis_sichuanensis GCA_021379005.1	99.53	0.94	5348817	50.3	nifD,nifK ,nifH	Rubisco-S, Rubisco-L	coxA,coxB	sat	-
Leptolyngbya_boryana GCF_002142475.1	99.41	1.18	6803468	47	nifD,nifK ,nifH	Rubisco-S, Rubisco-L	coxA,coxB	aprA,sat,sqr,sdo	-
*Leptolyngbya_cf.ectocarp*i *GCA_015207065.1*	98.1	1.4	6774485	49.4	-	Rubisco-S, Rubisco-L	coxA,coxB	aprA,cysC,sat,sqr,sdo	-
*Moorella_thermoacetica ATCC_39073 GCF_000013105.1_*	99.23	0	2628784	55.79	nifD	cooS,acsD	cydA,cydB	asrA,asrB,asrC,dsrD,dsrA ,dsrB,phsA	-
Nostoc_punctiforme PCC_73102 GCF_000020025.1	99.56	0.44	9059191	41.35	nifD,nifK ,nifH	Rubisco-S, Rubisco-L	coxA,coxB	aprA,cysC,sat,sdo	-
*Nostoc_sp. PCC_7107 GCF_000316625.1_*	99.26	0.33	6329823	40.36	nifD,nifK ,nifH	Rubisco-S, Rubisco-L	cydA,cydB,coxA,coxB	cysC,sat,sdo	-
Rippkaea_orientalis GCF_000021805.1	99.56	0.22	4787694	39.76	nifD,nifK ,nifH	Rubisco-S, Rubisco-L	coxA,coxB	cysC,sat,dsrD,sdo	-
Thermosynechococcus_vestitus GCA_003555505.2	100	0.12	2650294	53.3	-	Rubisco-S, Rubisco-L	cydA,cydB,coxA,coxB	sat,sqr,sdo	-

Lowest Median Highest

Statistics without reference	Calothrix thermalis	Nostoc edaphicum	Anabaena lutea	Hillbrichtia pamiria	Thermoleptolyngbya hindakiae	Amphirytos necridicus
# contigs	385	217	72	58	39	199
# contigs (>= 0 bp)	385	217	72	58	39	199
# contigs (>= 1000 bp)	385	217	72	58	39	199
# contigs (>= 10000 bp)	65	157	58	48	33	66
# contigs (>= 100000 bp)	22	14	18	15	20	25
# contigs (>= 1000000 bp)	0	0	0	0	0	0
Largest contig	573 875	330 772	455 092	426 492	635 415	297 150
Total length	9 123 886	8 166 504	5 748 367	4 787 020	6 630 859	6 593 453
Total length (>= 0 bp)	9 123 886	8 166 504	5 748 367	4 787 020	6 630 859	6 593 453
Total length (>= 1000 bp)	9 123 886	8 166 504	5 748 367	4 787 020	6 630 859	6 593 453
Total length (>= 10000 bp)	7 669 338	7 852 613	5 686 107	4 737 999	6 599 164	5 965 044
Total length (>= 100000 bp)	6 344 213	2 620 203	4 001 541	3 227 321	5 933 341	4 275 253
Total length (>= 1000000 bp)	0	0	0	0	0	0
N50	241 165	73 557	169 088	184 907	357 711	135 684
N75	55 470	32 761	88 444	92 228	183 806	74 546
L50	12	31	10	10	7	17
L75	29	74	22	19	14	33
GC (%)	41.28	41.53	39.3	47.53	43.27	50.67
**Mismatches**						
# N's	3092	3377	1520	1203	3091	1605
# N's per 100 kbp	33.89	41.35	26.44	25.13	46.62	24.34

Figure S2. Detailed statistics about the length of contigs included in the 6 target genomes assembly.

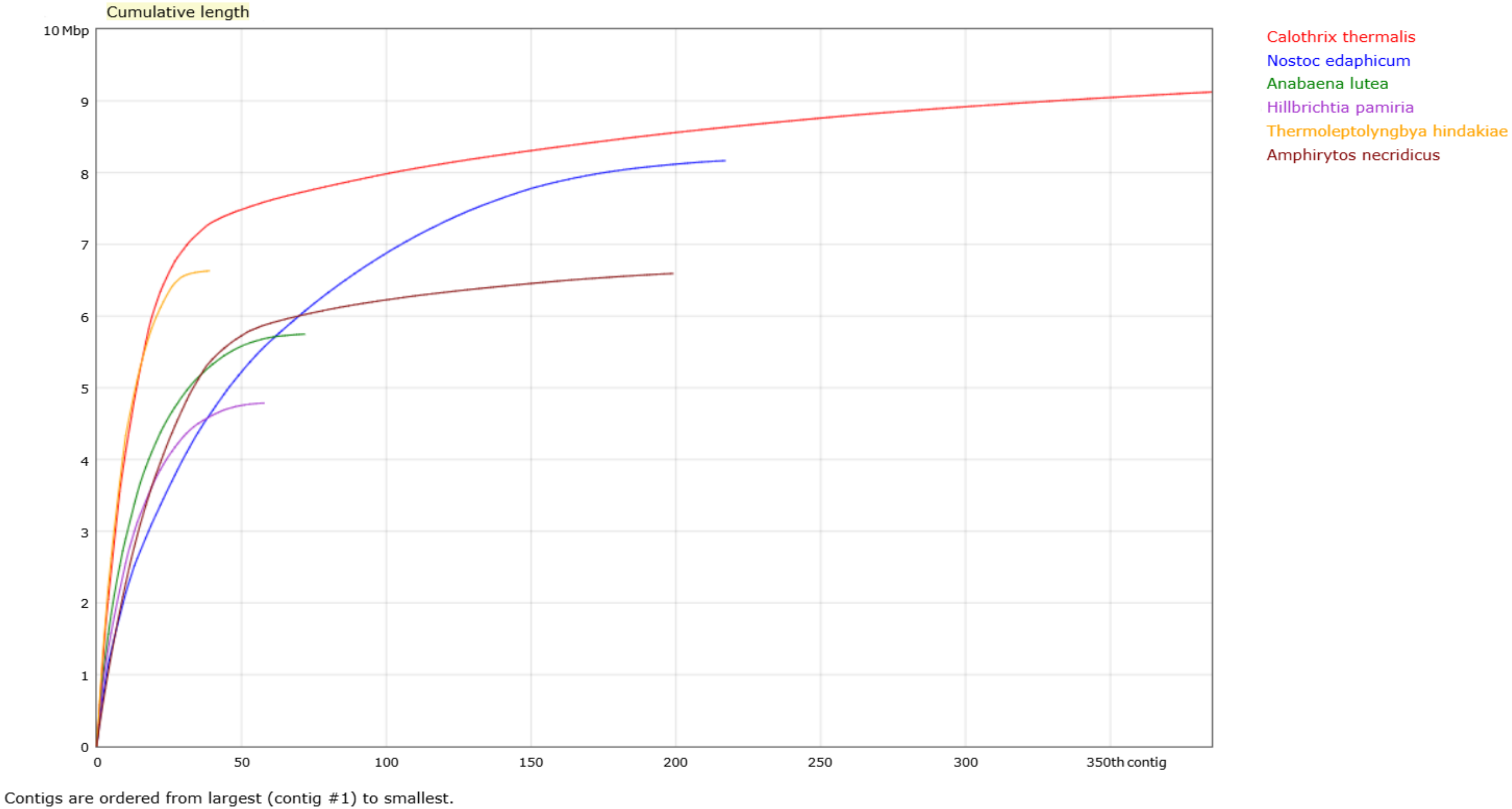

Figure S3. Number of contigs comprising each of the assembled genomes

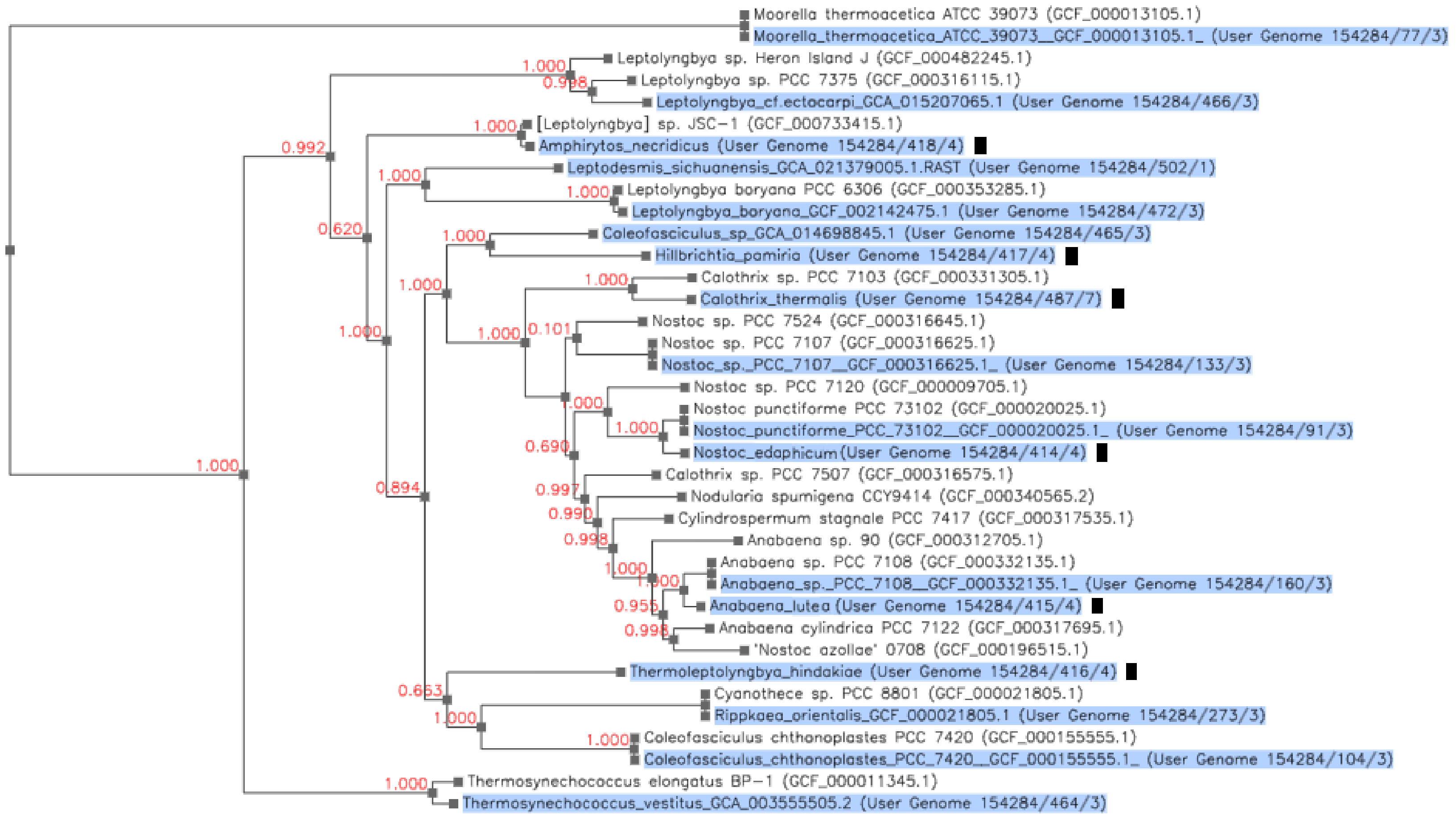

Figure S4. Phylogenomic tree of 40 cyanobacterial genomes, excluding the strains sequenced in this study and selected thermophilic reference genomes. Genomes highlighted in blue correspond to the isolates sequenced in this study and the thermophilic reference genomes used for comparative phylogenetic placement. Based on algaebase.org (2025). Highlighted in blue are genomes taken into consideration in this study.

Citation:

Guiry GM. 2023. AlgaeBase. World-wide electronic publication, National University of Ireland, Galway. https://www.algaebase.org; searched on 20 June 2025

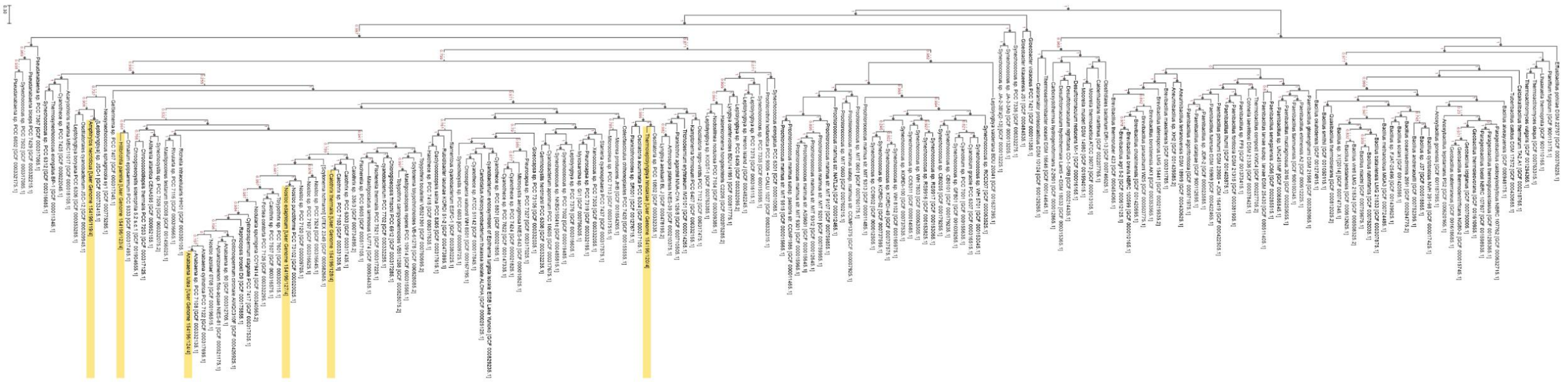

Figure S5. Phylogenomic tree based on complete cyanobacterial genomes used in this study and 200 reference genomes. Genomes assembled in this study are marked in yellow. The genomes included in this analysis ranged in size from 4.79 to 9.12 Mb, providing a representative set for phylogenetic placement of the newly sequenced strains

Table S2: Accession numbers for all assembled genomes deposited under ENA project number PRJEB93759

Accession	BioSample	Title	Organism	Tax id
**ERS29129821**	SAMEA121633933	NE8	Nostoc edaphicum	264686
**ERS29129820**	SAMEA121633932	TH23	Thermoleptolyngbya hindakiae	2931968
**ERS29129819**	SAMEA121633931	AL11	Anabaena lutea	212350
**ERS29129818**	SAMEA121633930	HP23	Hillbrichtia pamiria	2931972
**ERS29129817**	SAMEA121633929	CT23	Calothrix thermalis	373981
**ERS29129816**	SAMEA121633928	AN23	Amphirytos necridicus	2931970

Table S3. List of secondary metabolites discovered using antiSMASH

Region	Type of metabolite	From	To	Most similar cluster	Similarity
**AN**	***Amphirytos necridicus***				
9.1	spliceotide, PR-containing	241003	256187	PcPA	33%
13.1	lanthipeptide-class-V	20582	62927		
14.1	RiPP-like	63180	742444		
18.1	terpene	110174	131103		
24.1	NRPS-like, hglE-KS, T1PKS	24228	84674	heterocyte glycolipids	71%
39.1	RRE-containing, lassopeptide	1	18732		
**HP**	***Hillibrichtia pamiria***				
5.1	RRE-containing	95632	115964		
10.1	terpene	113182	134117		
23.1	cyanobactin	1	12799		
27.1	NRPS-like	5375	49274		
**TH**	***Thermoleptolyngbya hindakiae***				
2.1	RiPP-like	195305	206150		
2.2	lanthipeptide-class-II	306177	329380		
2.3	NRP-metallophore, NRPS, T1PKS	545425	620671	anachelin	35%
3.1	LAP	111769	152730	O&K-antigen	3%
7.1	terpene	6749	27675		
7.2	cyanobactin	129955	151874		
8.1	microviridin	190404	210703	microviridin 1688/1739/1748	25%
8.2	NRPS	224493	273426		
9.1	NRPS	115517	163538		
10.1	RRE-containing	129670	149918		
12.1	NRPS-like	214912	260062		
**CT**	***Calothrix thermalis***				
2.1	terpene	157450	181030		
2.2	NRPS, T1PKS, NRP-metallophore	271407	369356	anachelin	35%
2.3	NRPS-like, T1PKS	371066	430806	puwainaphycin A/B/C/D	20%
3.1	terpene	37935	58744		

3.2	hglE-KS, T1PKS	199004	251675	heterocyte glycolipids	85%
7.1	redox-cofactor	6669	28767		
7.2	terpene, LAP	249209	277636		
16.1	NRPS, mycosporine-like	118974	166761	hexose-palythine-serine/hexose-shinorine	57%
21.1	terpene	81171	92389		
25.1	lanthipeptide-class-V	20878	63231	hassallidin C	6%
25.2	spiceotide, RRE-containing	72713	95164	PcpA	33%
27.1	lanthipeptide-class-II, proteusin	49982	75824	landornamide A	22%
47.1	NRPS-like	1	13912		
**NE**	***Nostoc edaphicum***				
1.1	spiceotide, RRE-containing	29513	51392		
2.1	terpene	17609	39855	geosmin	100%
2.2	mycosporine-like	83961	127738	hexose-palythine-serine/hexose-shinorine	42%
2.3	NRPS	258184	304102	thaxteramide A1/A2/B1/B2	13%
7.1	spiceotide, RRE-containing	71971	94384		
8.1	lanthipeptide-class-II	136918	158021		
12.1	terpene	145288	159162		
15.1	terpene	36229	58145		
20.1	lanthipeptide-class-V	17279	59639		
26.1	hglE-KS, T1PKS	33019	81990	heterocyte glycolipids	100%
27.1	NRPS, T1PKS, NRP-metallophore	14000	80897	scytocyclamide A/B/B3/C/A2/B2	61%
34.1	NRPS, T1PKS, microviridin	1	55078	nostopeptolide A2	100%
39.1	terpene	1	11700		
41.1	terpene	24178	45143		
44.1	hglE-KS, T1PKS	14184	56899	heterocyte glycolipids	57%
55.1	NRPS, micoviridin	1	44153	nostocyclopeptolide A2	57%
56.1	NRPS-like, T1PKS, NRPS	1	43812	nostopeptolide A1/1052	46%
63.1	NRPS-like	5045	35316		
89.1	NRPS	1	26021	scytocyclamide A/B/B3/C/A2/B2	40%
99.1	lanthipeptide-class-V	1	23090		
129.1	T1PKS, NRPS	1	15258	hapalosin	40%

147.1	lanthipeptide-class-II	1	10530		
152.1	phosphonate	2018	12957		
178.1	NRPS-like, T1PKS	1	4933	jamaicamideA/B/C	15%
**AL**	***Anabaena lutea***				
2.1	lanthipeptide-class-V, spiceotide, RRE-containing	92843	135136	PcpA	33%
3.1	LAP	118381	142209	muscorideA/B	18%
3.2	hglE-KS, T1PKS	254991	305064	heterocyst glycolipids	57%
4.1	NRPS	97839	147729	puwainaphycin A/B/C/D	50%
4.2	NRPS, T1PKS, NRP-metallophore	185369	265106		
5.1	terpene	202919	223851		
9.1	NRPS-like, T1PKS, NRPS	37841	99463		
10.1	thipeptide, LAP	75833	105405		
13.1	lanthipeptide-class-V	80484	122785		
19.1	LAP	49160	71466		
22.1	hglE-KS, T1PKS	6335	58385	heterocyte glycolipids	100%
24.1	lanthipeptide-class-V	1	33710		
75.1	terpene	1	14323		
75.2	terpene	42927	64837		
75.3	NRPS	80897	120048		

Table S4. Summary of most important conclusions from comparative genomic analyses of six newly assembled cyanobacterial genomes

Feature / Category	*Hillbrichtia pamiria* HP	*Thermoleptolyngbya hindakiae* TH	*Amphirytos necridicus* AN	*Calothrix thermalis* CT	*Nostoc edaphicum-related* NE	*Anabaena lutea-related* AL	Main Conclusions
**Genome size (Mbp)**	4.7	5.2	6.1	9.1	8.7	8.5	Genome size varies 4.7–9.1 Mbp; larger Nostocaceae genomes (CT, NE, AL) contain expansive gene content and secondary metabolite pathways.
**GC content (%)**	50	48	50	49	47	39	GC content ranges from 39–50%, potentially contributing to thermal genome stability.
**Respiration / Electron Transport**	cox present; cyd present	cox present; cyd present	cox present; cyd absent	cox present; cyd present	cox present; cyd present	cox present; cyd absent	AL and AN lack cytochrome bd oxidase (cyd), indicating functional divergence in aerobic respiration.
**Sulfur Assimilation**	cysC, sat, sqr, sdo present	Partial	Partial	Present	Present	Partial	Heterogeneous sulfur gene presence reflects adaptation to local sulfur availability.
**Calvin Cycle (RuBisCO)**	~1.0	~1.0	~1.0	~1.0	~1.0	~1.0	Carbon fixation machinery conserved across all genomes.
**TCA Cycle Completeness**	0.88	0.88	1	1	1	1	TCA cycle incomplete in A. necridicus (~0.63), complete in most genomes; reflects energy-efficient adaptation using alternative enzymes.
**Nitrogen Metabolism (nif genes)**	nifHDK complete	nifHDK complete	nifHDK complete	nifHDK complete	nifHDK complete	nifHDK complete	Nitrogen fixation conserved; heterocyte glycolipid genes present in Nostocaceae (NE, AL) indicate oxygen-tolerant fixation.
**Denitrification (nirB/D)**	Absent	Absent	Present	Present	Present	Present	Variable presence suggests lineage-specific denitrification capabilities.

**Feature / Category**	***Hillbrichtia pamiria*** **HP**	***Thermoleptolyngbya hindakiae*** **TH**	***Amphirytos necridicus*** **AN**	***Calothrix thermalis*** **CT**	***Nostoc edaphicum-related*** **NE**	***Anabaena lutea-related*** **AL**	**Main Conclusions**
**Photosystems / CCMs**	Reduced PS complexity, enhanced heterotrophic C-utilization	Expanded PS II, CCM	Expanded PS II, CCM	Complete PS I/II	Complete PS I/II	Complete PS I/II	Photosystems conserved; expanded PS II and CCMs in Leptolyngbya reflect adaptation to high-light environments; A. necridicus adapted to low-energy niches.
**Secondary Metabolite BGCs**	Low	Moderate	Low	High	High	High	BGC abundance generally correlates with genome size; AL retains many BGCs despite smaller genome, indicating selective retention.
**Toxins (mcy, cyr)**	None	cyr present	None	cyr present	mcy & cyr present	mcy present	Microcystin restricted to Nostocaceae; cylindrospermopsin detected in NE, CT, TH; A. necridicus and HP lack toxins.
**Other Bioactive Compounds (RiPPs, lanthipeptides)**	None	RiPP/lanthi-peptides present	None	RiPP/lanthi-peptides present	Cyanobactins, microviridins present	RiPP/lanthi-peptides present	Suggests antimicrobial and bioactive potential, highlighting pharmaceutical relevance.
